# Modeling of Textile Dye Removal from Wastewater Using Innovative Oxidation Technologies (Fe(II)/Chlorine and H_2_O_2_/Periodate Processes): Artificial Neural Network-Particle Swarm Optimization Hybrid Model

**DOI:** 10.1021/acsomega.2c00074

**Published:** 2022-04-15

**Authors:** Abdelhalim Fetimi, Slimane Merouani, Mohd Shahnawaz Khan, Muhammad Nadeem Asghar, Krishna Kumar Yadav, Byong-Hun Jeon, Mourad Hamachi, Ounissa Kebiche-Senhadji, Yacine Benguerba

**Affiliations:** † Laboratoire des Procédés Membranaires et des Techniques de Séparation et de Récupération, Faculté de Technologie, Université de Bejaia, 06000 Bejaia, Algeria; ‡ Laboratory of Environmental Process Engineering, Department of Chemical Engineering, Faculty of Process Engineering, University Constantine 3 − Salah Boubnider, P.O. Box 72, 25000 Constantine, Algeria; § Department of Biochemistry, College of Science, King Saud University, Riyadh 11451, Saudi Arabia; ∥ Department of Medical Biology, University of Québec at Trois-Rivieres, Trois-Rivieres, Québec G9A 5H7, Canada; ⊥ Faculty of Science and Technology, Madhyanchal Professional University, Ratibad, Bhopal 462044, India; # Department of Earth Resources and Environmental Engineering, Hanyang University, Seoul 04763, Republic of Korea; ∇ Department of Process Engineering, Faculty of Technology, University Ferhat ABBAS Setif-1, 19000 Setif, Algeria

## Abstract

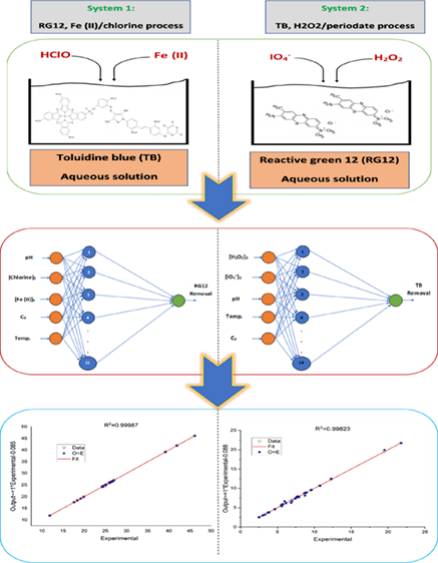

An efficient optimization technique based on a metaheuristic and an artificial neural network (ANN) algorithm has been devised. Particle swarm optimization (PSO) and ANN were used to estimate the removal of two textile dyes from wastewater (reactive green 12, RG12, and toluidine blue, TB) using two unique oxidation processes: Fe(II)/chlorine and H_2_O_2_/periodate. A previous study has revealed that operating conditions substantially influence removal efficiency. Data points were gathered for the experimental studies that developed our ANN-PSO model. The PSO was used to determine the optimum ANN parameter values. Based on the two processes tested (Fe(II)/chlorine and H_2_O_2_/periodate), the proposed hybrid model (ANN-PSO) has been demonstrated to be the most successful in terms of establishing the optimal ANN parameters and brilliantly forecasting data for RG12 and TP elimination yield with the coefficient of determination (R2) topped 0.99 for three distinct ratio data sets.

## 1 Introduction

To remove organic pollutants, nutrients, and other impurities, a typical wastewater treatment plant uses a variety of physical, chemical, and biological unit processes. Detritus that is solubilized by microorganisms may only be removed using natural treatment.^1,2^ Industrial effluents are sometimes more challenging because of high organic matter content, non-neutral pHs, salinity, or the inclusion of synthetic chemicals with long persistence and low biodegradability.^3^ This treatment chain is suitable for the majority of household wastewaters. One of the most common occurrences is wastewater from the textile industry. The color is still discernible at low concentrations (less than 1 ppm for some dyes).^4−7^ Toxic, carcinogenic, mutagenic, or teratogenic compounds are often found in textile wastewater. For the most part, the chromophore grouping of a dye is employed to sort it out. Although anthraquinone, xanthene, phthalocyanine, and sulfur are also utilized, most chemicals are azo (−N=N−) derivatives.^8^ Some substances may alter wastewater treatment facilities, leading to more stable and harmful organisms, or they may not change and remain unchanged. For decades, scientists have been working to create advanced oxidation processes (AOPs) that are more environmentally friendly.^9,10^ Hydroxyl radical ^•^OH, a potent oxidant (*E*° = 2,8 V) and highly reactive species to most organic contaminants in situ, is produced by AOPs. AOPs include the Fenton system (Fe(II)/H_2_O_2_), UV/H_2_O_2_, H_2_O_2_/O_3_, UV/O_3_, and UV/TiO_2_.^11^ An alternative to H_2_O_2_ and UV has recently been explored: the UV/chlorine procedure. It has been tried in a pilot or full-scale plant for water treatment, drinking water processing, and groundwater remediation.^12^ In this process, several free radicals, including ^•^OH, Cl_2_^•–^, and ClO^•^, are generated to collectively remove micropollutants at a much faster rate. Micropollutant elimination is an important goal for environmentalists and scientists alike. Utilizing multiple free radicals in a novel class of oxidation processes is one way to achieve this goal.^13^ As with UV/chlorine, UV/periodate acts as a multifree radical generator, producing radicals such as IO_3_^•^, IO_4_^•^, ^•^OH, IO_3_, O(^3^P), O_3_, and H_2_O_2_ for the removal of a variety of water contaminants.^14−17^

To integrate the results of experiments, mathematic models were regarded as appropriate. Kinetic modeling models have many constraints because of their complicated nature and nonlinearity, limiting the parameters.^18−27^ Scientists have put out fuzzy logic (FL) models, artificial neural networks (ANNs), and other ML techniques. The latter have been applied to processes in several studies.^28−36^ Machine learning (ML) offers practical solutions to solve challenging issues in various industrial applications.^4^ ML includes computer algorithms and statistical methods required for data-driven control, estimation, prediction, classification, or clustering.^37^

Although it is not practicable, complicated problems that were difficult to describe and analyze may now be appraised using these methods.^38^ Several ANNs, such as a multilayer perceptron, are based on the human brain (MLP). This rigorous mathematical model often used in ML may explain any nonlinear relationship between input and output sets. An ANN is a collection of neurons linked by two fundamental parameters (connection weights and thresholds).^39−41^ Neural network techniques such as Levenberg–Marquardt (LM), scaled gradient descent (SGD), and gradient descent with momentum (GDWM) are among the most often employed (GDM). Adaptation, learning, and generalization may occur even when working with nonlinear functions. Contrarily, the speed of convergence of BPNN algorithms is slow. Some metaheuristic optimization approaches, such as the genetic algorithm (GA), firefly algorithm (FA), ant colony optimization (ACO), particle swarm optimization (PSO), and differential evolution (DE), may be utilized to solve these problems. They help the ANN find more optimal solutions faster, increasing its overall efficiency. Techniques like PSO and GA illustrate this trend.^40,41^ To cope with the most complex and complicated issues in optimization, they are the most promising global optimization approaches.^42−46^

They have recently discovered two new oxidation methods for efficiently removing textile colors from wastewater.^47,48^ These two reactions, Fe(II)/chlorine and H_2_O_2_/periodate, have been discovered as multiple sources of free-radical oxidation of organic contamination. Cl^•^, ClO^•^, and Cl_2_^•–^ chlorine radicals have been implicated in the Fe(II)/Chlorine process,^38,39^ while IO_3_^•^ and IO_4_^•^ iodine radicals, as well as singlet oxygen (^1^O_2_), have been implicated in the H_2_O_2_/periodate system.^47,49^

- Fe(II)/chlorine process
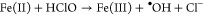
1
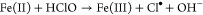
2
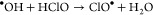
3
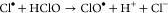
4
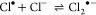
5
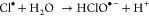
6
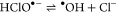
7
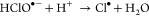
8
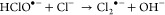
9
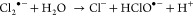
10
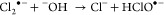
11
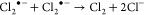
12
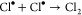
13
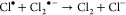
14
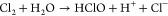
15
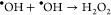
16
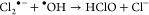
17
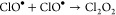
18
- H_2_O_2_/periodate
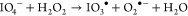
19
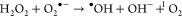
20
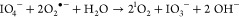
21
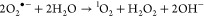
22
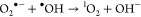
23
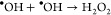
24
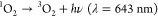
25
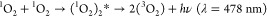
26
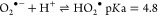
27
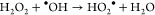
28
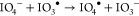
29
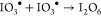
30
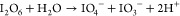
31
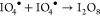
32
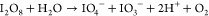
33
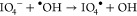
34

To degrade RG12 and TB textile colors fast, we used Fe(II)/chlorine and H_2_O_2_/periodate processes. Several operational variables, including as reagent dosages, solution temperature, and pH, were examined throughout a wide range of reaction times. Industrial applications need a modeling technique that maximizes the efficiency of both kinetic processes.

This work aimed to develop a hybrid model (ANN-PSO) for the case of the removal of RG12 and TB dyes from wastewater utilizing Fe(II)/chlorine and H_2_O_2_/periodate oxidation processes. The PSO metaheuristic optimization was combined with ANN to construct a feasible model for predicting and optimizing the removal of textile colors from wastewater effluent using Fe(II)/chlorine and H_2_O_2_/periodate, respectively. The model’s adaptability and durability were shown using the *R*^2^ coefficient and the root mean square error (RMSE) between the predicted and experimental datasets.

## 2 Experimental Data

Merouani et al.^47,48^ developed the ANN-PSO model by assessing the removal kinetics of RG12 and TB dyes from aqueous solutions under different experimental settings, utilizing the Fe(II)/chlorine and H_2_O_2_/periodate oxidation systems, respectively. The testing methodology and data are summarized in Text S1 in the Supporting Information.

For the Fe(II)/chlorine system, 146 datasets were collected from the experimental assessment of the removal kinetics of RG12 over time under various experimental factors such as solution pH (3–7.9), chlorine dosage (25–250 mM), Fe(II) initial concentration (5–100 M), initial RG12 concentration (*C*_0_: 10–100 mg/L), and liquid temperature (20–40 °C). For H_2_O_2_/periodate, 169 datasets were collected from the experimental assessment of TB removal kinetics over time under various experimental factors: initial H_2_O_2_ concentration (10–200 mM); initial periodate dosage (0.5–10 mM); initial solution pH (3–10.5); initial TB concentration (*C*_0_: 5–50 mg/L); and liquid temperature (10–50 °C).

## 3 Materials and Methods

### 3.1 ANN Methodology

Input (IL), output (OL), and intermediate or hidden (HL) layers (Figures S1 and S2 of the Supporting Information) are the three architectural levels of ANNs.^51,53,54^ Neurons (or nodes) are essential computer components used in parallel computing.^53−55^ Neurons work together to detect input data sets commonly seen.^56^
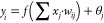
35*f* is the transfer function; *x_j_* is the neuron’s inputs; and *w_ij_* is the link between IL and HL (weights) and the HL’s threshold of *j* neuron.^50,53^ Constructing and training neuronal networks has as its primary goal the minimization of the objective function (or fitness function), which in turn leads to better predictions for new input.^52,57^ The latter compares the output and experimental data sets to determine how well the network operates. The fitness (error) function may thus be written as follows:
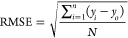
36*N* is the number of experiments, and *y_i_* and *y_o_* are the calculated and experimental data, respectively.^57^

### 3.2 Particle Swarm Optimization

It is a nature-inspired evolutionary computing technology based on the movement and intelligence of swarms, such as ants and birds.^38^ Kennedy and Eberhart created PSO in 1995 as a resilient stochastic optimization approach. Numerous optimization problems, such as function optimization, fuzzy control, and pattern recognition, have been solved using this approach.^58,59^

Arbitrary particles, also known as solutions, are supported by the PSO algorithm. The search space (or the state space) is transformed into a swarm that seeks only the most advantageous options. The PSO training iteration uses the experience of individual particles and those around them to adjust their position and speed.
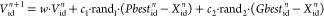
37
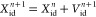
38*V*_id_^*n* + 1^ is the new velocity; *Pbest*_id_^*n*^ is the best position of the particle during training; *Gbest*_id_^*n*^ is the best position among all the particles in the swarm during the training iteration. The cognitive influence is *Pbest*_id_^*n*^ – *X*_id_^*n*^ and the social influence is *Gbest*_id_^*n*^ – *X*_id_^*n*^. *c*_1_ and *c*_2_ (acceleration constants) are the cognition and social weights, respectively.

*w* is the inertia weight (or inertia constant).^60^ rand_1_ and rand_2_ generate random values ranging from 0 to 1. An example of the standard flow chart for the PSO approach may be seen in Figure S3 (Supporting Information).

The fundamental idea behind the PSO approach is that each particle is accelerated toward its *Pbest*_id_^*n*^ and *Gbest*_id_^*n*^ positions at each iteration.

When searching for the *k*-dimension and *m*-size of the acceleration, each particle may be represented as follows: *X_i_* = (*x*_*i*1_, *x*_*i*2_...*x_iD_*)^*T*^; *V_i_* = (*v*_*i*1_, *v*_*i*2_...*v_iD_*) (see Figure S4 in the Supporting Information).

### 3.3 PSO Approach

Analysis of state space is carried out by using a collection of particles. Weights and thresholds are stored in each particle for later adjustment.^39^

Figure S5 of the Supporting Information shows the crucial phases of the ANN-PSO hybrid algorithm.

1. Importing experimental data.
2. Define ANN structure, weights, and thresholds.
3. Compute the number of weights and thresholds:
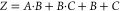
39
  *A*, *B*, and *C* are for IL, HL, and OL, respectively.
4. Initialize parameters.
5. Calculate the error, *Pbest* and *Gbest*.
6. Adjust the location and velocity (eqs 37 and 38).
7. If *f*(*X*_id_^*n* + 1^) < *f*(*Pbest*_id_^*n*^) Then *Pbest*_id_^*n* + 1^ = *X*_id_^*n* + 1^
  Else *Pbest*_id_^*n* + 1^ = *Pbest*_id_^*n*^
8. If *f*(*X*_id_^*n* + 1^) < *f*(*Gbest*_id_^*n*^) Then *Gbest*_id_^*n* + 1^ = *X*_id_^*n* + 1^
  Else *Gbest*_id_^*n* + 1^ = *Gbest*_id_^*n*^
9. The ANN parameters’ results are shown (weights, thresholds).

### 3.4 Database and Termination Criteria

Table 1 illustrates how the 146 experimental datasets for the Fe(II)/chlorine system and the 169 H_2_O_2_/periodate system were split into 70% training, 15% testing, and 15% validation. Initial solution pH, initial chlorine (or H_2_O_2_) concentration, initial Fe(II) (or periodate) concentration, initial RG12 (or TB) concentration, and initial liquid temperature are all factors in the IL. The removal efficiency of RG12 (or TP) is saved in the OL (see Table 2).

**Table 1:** Data Distribution of RG12 and TB Removal into Three Sets

divide rand	percentage (%)	divide data
*system 1: RG12, Fe(II)/chlorine process*		
train ratio	70	102
test ratio	15	22
validation ratio	15	22
*system 2: TP, H_2_O_2_/periodate process*		
train ratio	70	119
test ratio	15	25
validation ratio	15	25

**Table 2:** Range of All Parameters

parameters	minimum value	maximum value
*system 1: RG12, Fe(II)/chlorine process*		
*IL*		
pH	3	8
[chlorine]_0_ (μM)	25	1000
[Fe(II)]_0_ (μM)	0	100
*C*_0_ (mg/L)	10	100
temp. (°C)	10	40
*OL*		
RG12 removal (mg/L)	3.59	44.26
*system 2: TB, H_2_O_2_/periodate process*		
*IL*		
[H_2_O_2_]_0_ (mM)	3	8
[IO_4_^–^]_0_ (mM)	25	1000
pH	3	11
temp. (°C)	10	50
*C*_0_ (mg/L)	5	50
*OL*		
TB removal (mg/L)	2.04	22

To evaluate or confirm the algorithm’s halting conditions, the stopping criteria of this algorithm is considered or validated when the maximum number of iteration or minimum RMSE is attained.

## 4 Results and Discussion

It is feasible to assess whether or not the mathematical model’s predictions stand up under investigation using experimental data. Training the ANN-PSO hybrid model necessitates altering the number of neurons in the intermediate. Transfer functions are sigmoid in both the HL and OL. Most optimization strategies use it as a fitness function when assessing the proposed model’s training performance. We are searching for parameters (weights and thresholds) that minimize the objective function (RMSE) between the anticipated outcomes of our models and the experimental datasets.

### 4.1 Fe(II)/Chlorine System

The five parameters in the IL were initial solution pH (from 3 to 7.9), initial chlorine concentration (from 25 to 1000 M), initial Fe(II) concentration (from 0 to 100 M), initial RG12 concentration (from 10 to 100 mg/L), and liquid temperature (from 10 to 40 °C). The OL includes RG12 removal (3.59–44.26 mg/L).

The best optimal parameters were *c*_1_ and *c*_2_, of 1.25 and 2.5, respectively. *w* = 0.15; maximum number of iterations = 4500, and swarm size is 15:

- Fifty-five weights, *w_ij_* (11 × 5) correlating IL with HL.
- Eleven weights, *w*_*j*1_ correlating HL with OL.
- Eleven thresholds, θ_*j*_ for HL’s neurons.
- One threshold θ_*j*_ for the OL (see Table 3).

**Table 3:** Optimal ANN-PSO Parameters (*System 1: RG12, Fe(II)/Chlorine Process*)

*w_ij_*	1	2	3	4	5	6	7	8	9	10	11
1	–1.91	1.395	–0.742	0.878	–0.695	–1.91	1.547	–0.42	0.419	1.91	0.408
2	1.709	–0.568	–0.803	1.91	–1.691	–0.101	1.344	1.285	0.839	–1.896	–1.004
3	–0.318	–0.817	0.333	0.073	–1.054	–1.91	0.516	–1.91	–0.216	–1.08	0.879
4	1.67	1.69	–1.91	–1.157	1.91	–1.91	–1.91	0.291	1.91	–1.231	0.307
5	–1.239	0.936	–0.122	–1.503	0.994	1.91	–1.91	0.463	1.91	–0.42	–1.91
*w*_1*j*_	1	2	3	4	5	6	7	8	9	10	11
1	1.049	–1.446	–0.993	1.615	0.436	–0.271	–0.357	–1.91	0.822	0.992	–1.727
θ_*j*_	1	2	3	4	5	6	7	8	9	10	11
1	–1.097	–0.193	–1.447	1.8	–1.734	0.669	–0.585	–1.293	0.855	1.119	1.91
θ_1_	–1.097										

**Figure 1:**
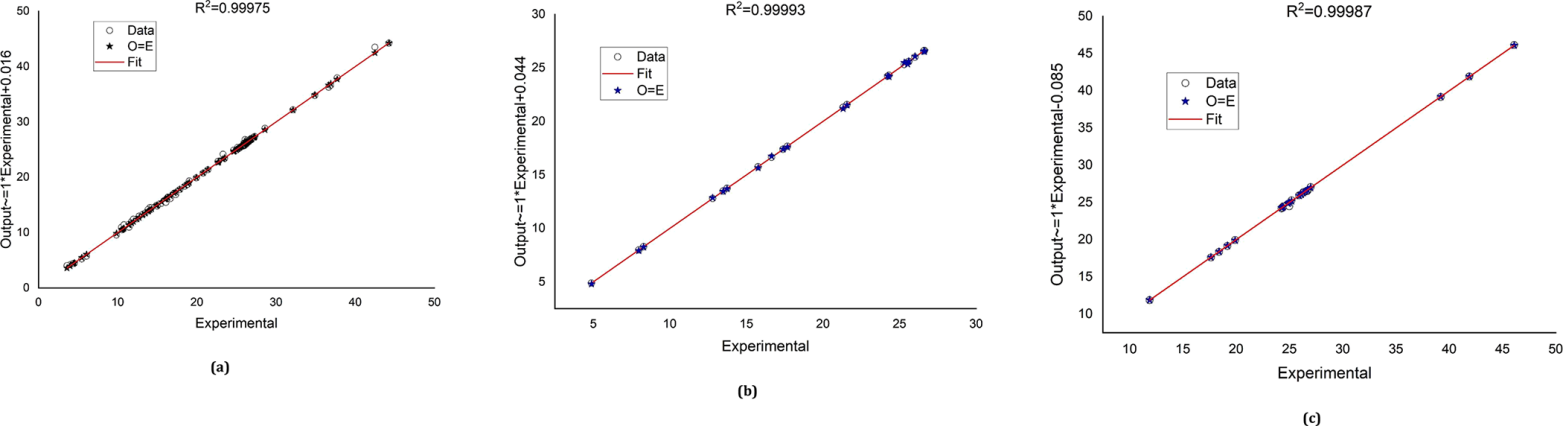
Regression plot of the output (ANN-PSO) and experimental data sets (System 1: RG12, Fe(II)/chlorine process): (a): training, (b): testing, and (c): validation.

Consequently, a 5:11:1 network (three layers) is the ultimate architectural network (i.e., five nodes in the IL, one HL with eleven nodes, and one node in the OL, respectively). After each training iteration, two variables, *Pbest* and *Gbest*, determine how each solution changes its position and velocity. The RMSE objective function assesses the model’s prediction ability by minimizing the difference between the actual and predicted data sets. As seen in Figure 1a, the ANN-PSO hybrid model presents and updates parameters (weights and thresholds) following its objective function, determining the output datasets throughout training. In Figure 1b, it can be seen how a network’s performance is evaluated when training is completed using a network test. Data sets for RG12 removal from wastewater treatment concentrations are forecasted using network validation in Figure 1c.

Figure 1 demonstrates the network output from the proposed mathematical ANN-PSO model, and the experimental data sets for the three stages (training, testing, and validation) generated using MATLAB software. The practical data sets were classified into three types: training data sets (70%), testing data sets (15%), and validation data sets (15%). For the three phases, the coefficients of determination (*R*^2^) are 0.99975, 0.99993, and 0.99987. The RMSE for each stage (training, testing, and validation) is 0.00181, 0.00084, and 0.00129, respectively. These statistics demonstrate strong performance across all data sets, with (*R*^2^) values closer to unity and an RMSE less than 0.0012. This shows that all data fall along a 45-degree line with a slope = 1. The network outputs generated by the ANN-PSO hybrid model and relevant experimental data sets have the same linear connection (perfect correlation, “Perfect fit”).

### 4.2 H_2_O_2_/Periodate System

The initial H_2_O_2_ concentration (10–200 mM), initial IO_4_^–^ concentration (from 0.5 to 10 mM), initial solution pH (3–11), liquid temperature (10–50 °C), and initial TB concentration (5–50 mg/L) were the five parameters in the IL. The elimination quantity in the OL is 2.04–22 mg/L.

Table 4 shows the most optimum parameters of the ANN model derived using the PSO method (*c*_1_ and *c*_2_ equal to 1.25 and 2.5, respectively, *w* = 0.25, a maximum number of iterations =4500, and swarm size = 15).

**Table 4:** Optimal Weights and Thresholds of ANN Using the PSO Algorithm (*System 2: TP, H_2_O_2_/Periodate Process*)

*w_ij_*	1	2	3	4	5	6	7	8	9	10	11	12	13	14
1	–0.770	0.900	–0.395	1.310	1.310	–1.163	0.771	1.310	–0.941	–0.317	–0.182	1.310	1.163	–1.310
2	0.193	1.310	–0.549	–1.310	0.699	0.956	0.546	0.763	–1.116	0.955	1.214	0.696	–0.217	0.288
3	–1.020	0.487	0.178	–1.310	0.706	–0.273	–0.597	0.259	–1.310	0.682	0.274	0.485	–1.310	–1.021
4	–0.432	1.031	–1.910	1.310	1.310	–0.035	0.544	0.603	–1.310	0.064	–0.422	–0.355	–1.310	0.310
5	0.252	1.310	–1.310	0.149	–1.310	0.751	–0.163	–0.435	–0.908	–0.358	0.235	–0.287	0.521	0.212
*w_1j_*	1	2	3	4	5	6	7	8	9	10	11	12	13	14
1	–1.067	0.132	–1.190	0.115	–0.059	–0.886	–1.310	–1.310	–1.310	–0.057	0.938	0.846	0.216	1.310
θ_*j*_	1	2	3	4	5	6	7	8	9	10	11	12	13	14
1	–1.094	0.965	–1.310	–1.197	–1.310	0.261	–1.261	0.391	1.310	1.310	1.300	0.256	–0.623	–0.612
θ_1_	–1.094													

They are distributed as follows:

- *w_ij_* (70 = 14 × 5) correlating IL with HL.
- *w*_*j*1_ (14) correlating HL with OL.
- θ_*j*_ (14) for HL’s neurons.
- θ_1_ (1) for OL.

The final architectural network is three layers: (5:14:1) network.

**Figure 2:**
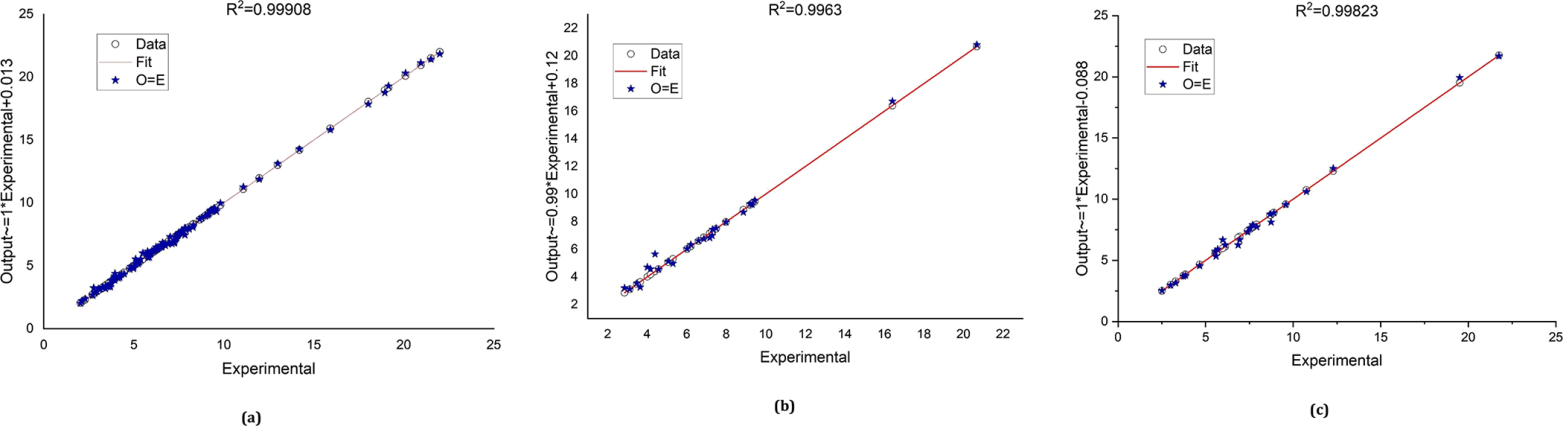
Regression plot of the output (ANN_PSO) and experimental data sets (System 2: TP, H2O2/periodate process): (a): training, (b): testing, and (c): validation.

Figure 2 depicts the proposed mathematical ANN-PSO model’s network output and the associated experimental data sets for the system H_2_O_2_/period throughout the three stages (training, testing, and validation) using MATLAB software. The practical data sets were classified into three types: training data sets (70%), testing data sets (15%), and validation data sets (15%).

The coefficient of determination, *R*^2^, was 0.99908, 0.9963, and 0.99823 for the three stages, respectively. The RMSE values for the three stages (training, testing, and validation) were 0.00174, 0.00350, and 0.00273, respectively. It is determined that the correlation is perfect because *R*^2^ is near one and RMSE values are less than 0.0018.

**Figure 3:**
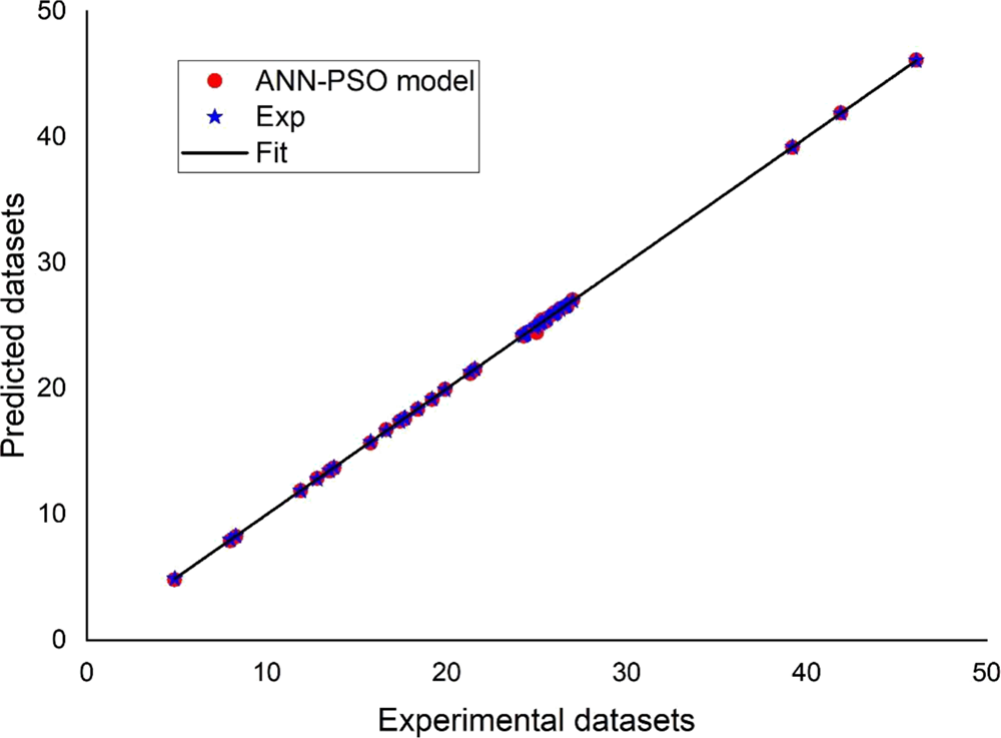
ANN-PSO-predicted datasets against experimental data sets (*System 1:* RG12, Fe(II)/chlorine process).

**Figure 4:**
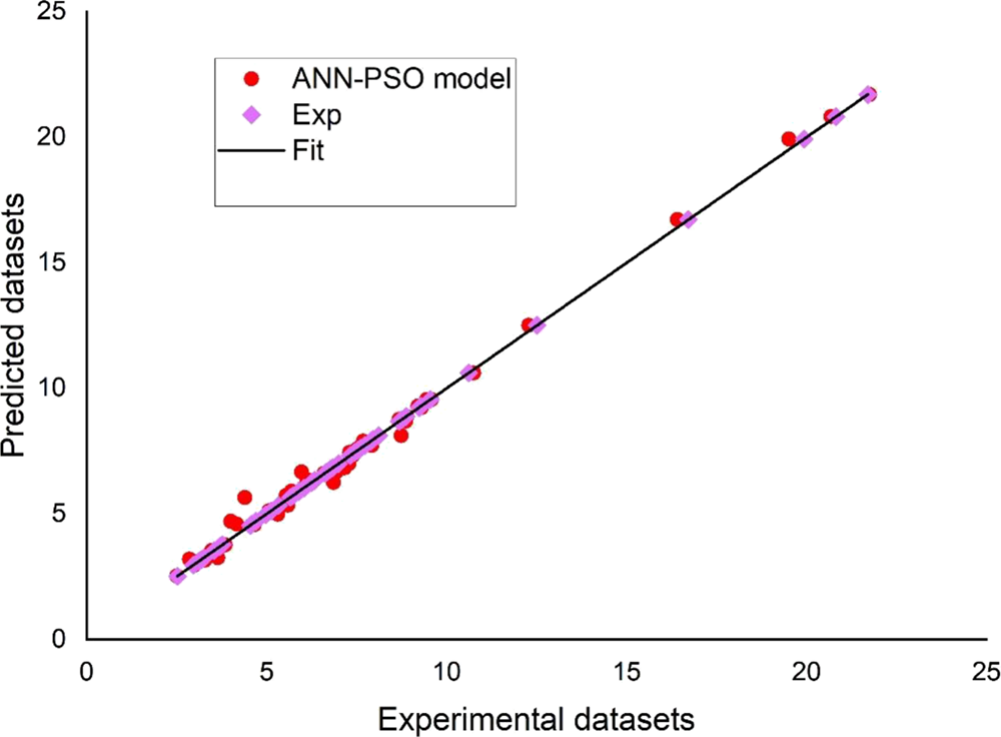
Plot of ANN-PSO predicted datasets against experimental data sets (*System 2:* TP, H_2_O_2_/periodate process).

Figures 3 and 4 compare the numerically simulated RG12 and TB removal (i.e., RG12 removal and TB Removal datasets) to the experimental data sets. The best fitting of the experimental data was also obtained for the two cases, revealing the ability of the ANN-PSO model toward predicting RG12 and TB removal.

For new RG12 data sets, Fe(II)/chlorine and H_2_O_2_/periodate processes were predicted more precisely using the ANN-PSO model. Table 5.

**Table 5:** Comparison of ANN-PSO Results Obtained for Both Systems

models	ANN model design	*R*^2^			RMSE		
		training	testing	validation	training	testing	validation
ANN-PSO: system 1^a^	05/11/2001	0.99975	0.99993	0.99987	0.00181	0.00084	0.00129
ANN-PSO: system 2^b^	5-14-1	0.99908	0.9963	0.99823	0.00174	0.0035	0.00273

a RG12, Fe(II)/chlorine process.

b TP, H_2_O_2_/periodate process.

It can be concluded that the experimental and simulated findings agreed very well.

## 5 Conclusions

This work investigates the use of a new approach based on an ANN and PSO method to remove RG12 and TB dyes from wastewater utilizing Fe(II)/chlorine and H_2_O_2_/periodate oxidation processes. The ANN training function adjusts the weights and threshold values according to the PSO approach. The use of MATLAB software carried out the results. The coefficient of determination (*R*^2^) from the ANN-PSO hybrid mathematical model topped 0.99 for three distinct ratio data sets from two independent systems. The proposed ANN-PSO model effectively predicts new RG12 and TB removal data sets with high (*R*^2^) and low RMSE values.

## Supporting Information Available

Experimental and data collection, different experimental runs for the removal of RG12 (mg/L) using the Fe(II)/chlorine oxidation system, different experimental runs for the removal of TB (mg/L) using H2O2/periodate oxidation system, ANN topology, standard flow chart of the PSO algorithm, concept of changing a particle’s position in PSO, and flow chart of the ANN-PSO hybrid mathematical model (PDF)
