# Supplementary material for: Modeling of Textile Dye Removal from Wastewater Using Innovative Oxidation Technologies (Fe(II)/Chlorine and H2O2/Periodate Processes): Artificial Neural Network-Particle Swarm Optimization Hybrid Model
Source: ACS Omega. 2022 Apr 15;7(16):13818–25. doi: 10.1021/acsomega.2c00074 (PMC9088958; doi:10.1021/acsomega.2c00074)
Supplement: Supplementary file 1 — ao2c00074_si_001.pdf [file ao2c00074_si_001.pdf]

Supporting Information:

Modeling of Textile Dyes Removal from Wastewater Using Innovative Oxidation Technologies (Fe (II)/Chlorine and H2O2/Periodate Processes): Artificial Neural Network-Particle Swarm Optimization (ANN-PSO) Hybrid Model

*Abdelhalim FETIMI [1], Slimane MEROUANI [2], Mohd Shahnawaz Khan [3], Muhammad Nadeem Asghar [4], Krishna Kumar Yadav [5], Byong-Hun Jeon [6], Mourad HAMACHI [1], Ounissa KEBICHE-SENHADJI [1], Yacine BENGUERBA[7, *]*

[1] *Laboratoire des Procédés Membranaires et des Techniques de Séparation et de Récupération, Faculté de Technologie, Université de Bejaia, 06000 Bejaia, Algeria ; abdelhalim.fetimi@univ-bejaia.dz (A. F) ; hamachi-m@hotmail.fr (H. M) ; kebiche_anissa@yahoo.fr (K. S. O.)*

[2] *Laboratory of Environmental Process Engineering, Department of Chemical engineering, Faculty of Process Engineering, University Constantine 3 - Salah Boubnider, P.O. Box 72, 25000 Constantine, Algeria; s.merouani@yahoo.fr*

[3] *Protein Research Chair, Department of Biochemistry, College of Science, King Saud University, Riyadh 11451, Saudi Arabia; moskhan@ksu.edu.sa*

Department of Medical Biology, University of Québec at Trois-Rivieres, Trois-Rivieres, Québec G9A 5H7, Canada; Muhammad.Nadeem.Asghar2@uqtr.ca

[5]*Faculty of Science and Technology, Madhyanchal Professional University, Ratibad, Bhopal, 462044, India; envirokrishna@gmail.com*

[6]*Department of Earth Resources and Environmental Engineering, Hanyang University, Seoul 04763, Republic of Korea; bhjeon@hanyang.ac.kr*

[7]*Laboratoire des Matériaux Polymères Multiphasiques, LMPMP, Université Ferhat ABBAS Sétif-1, 19000 Sétif, Algeria ; benguerbayacine@yahoo.fr*

***Corresponding author** (Y. BENGUERBA) benguerbayacine@yahoo.fr, Tel. +213556850449

Text S1: Experimental and data collection

C.I. reactive green 12 (abbreviation: RG12; CAS number: 12225-80-8; molecular formula: $C_{60}H_{29}Cl_3N_{16}NiO_{21}S_7.H_2O$, molecular weight: 1837.7 g mol^{-1}) and Toluidine Blue (abbreviation: TB; CAS number: 6586-04-5; molecular formula: $C_{15}H_{16}ClN_3S·0.5ZnCl_2$, molecular weight: 373.97 g mol^{-1}) were the pollutants models of the *Merouani's* experimental projects.[47, 48]. Both dyes are widely used in textile industries; they are toxic and can cause mutagenic effects.[47, 48].

For both systems (Fe(II)/chlorine and H_2O_2/periodate), degradation experiments were all conducted in batch mode (in a Pyrex cylindrical cell) using a fixed operational solution volumes (i.e., 200 mL for Fe(II)/chlorine system and 100 mL for the H_2O_2/periodate system), which were prepared using distilled water (water matrix for both studies). In all runs, treated solutions were open to air and their initial pHs were adjusted using H_2SO_4 or NaOH.

The concentration of dyes was followed spectrophotometrically at fixing time intervals using a Jenway UV-Vis. Spectrophotometer. Because the degradation rate is too fast, due to instantaneous reaction between Fe(II) and chlorine, in one part, and H_2O_2 and periodate, in another part, the dyes concentration during the treatment drops immediately from the initial value (C_0) to a fixed value ($<C_0$) at the first minute of the reaction (no change was observed above 1 min). Because of this reaction scenario, the removal efficiencies reported in Tables S2 and S2 (collecting the datasets over a range of operational parameters) is calculated after the first minute of the treatment (contact between reagents in the reactor).

For the system Fe(II)/chlorine, a total number of 146 datasets (Table S1) was collected from the experimental assess of the RG12 removed amount (in mg/L) under different experimental factors of solution pH (3-8), chlorine dosage (25-250 mM), Fe(II) initial concentration (5-100 μM), initial RG12 concentration (C_0: 10-100 mg/L) and liquid temperature (20-40 °C).

For the system, H_2O_2/periodate, A total number of 169 datasets (Table S2) was collected from the experimental assess of the TB removal (in mg/L) under different experimental factors of initial H_2O_2 concentration (10-200 mM), initial periodate dosage (0.5-10 mM), initial solution pH (3-10.5), initial TB concentration (C_0: 5-50 mg/L) and liquid temperature (10-50 °C).

TABLE S1. Data sets extracted from the different experimental runs for the removal of RG12 (mg/L) by Fe(II)/chlorine oxidation system under different operational conditions of solution pH (3-7.9), chlorine dosage (25-250 mM), Fe(II) initial concentration (5-100 µM), initial RG12 concentration (C_0: 10-100 mg/L) and liquid temperature (20-40 °C).

pH	[chlorine]$_0$ (µM)	[Fe(II)]$_0$ (µM)	C_0 (mg/L)	Temp.(°C)	RG12 Removal (mg/L)
3	250	25	30	20	26,02
3,5	250	25	30	20	25,36
4	250	25	30	20	25,15
4,5	250	25	30	20	25,69
5	250	25	30	20	24,75
5,5	250	25	30	20	22,77
6	250	25	30	20	19,05
6,5	250	25	30	20	14,10
6,70	250	25	30	20	12,44
6,75	250	25	30	20	11,75
6,8	250	25	30	20	11,39
6,85	250	25	30	20	11,45
6,9	250	25	30	20	10,57
7	250	25	30	20	9,85
7,5	250	25	30	20	6,08
7,6	250	25	30	20	5,47
7,8	250	25	30	20	4,54
7,9	250	25	30	20	3,98
8	250	25	30	20	3,59
5	25	25	30	20	13,17
5	35	25	30	20	13,93
5	40	25	30	20	14,29
5	45	25	30	20	14,89
5	50	25	30	20	15,55
5	60	25	30	20	16,30
5	65	25	30	20	16,35
5	75	25	30	20	17,14
5	85	25	30	20	17,85
5	95	25	30	20	18,50
5	100	25	30	20	18,85
5	150	25	30	20	21,40
5	200	25	30	20	23,50
5	250	25	30	20	24,92
5	400	25	30	20	26,78
5	500	25	30	20	26,93
5	600	25	30	20	26,20
5	700	25	30	20	26,23
5	800	25	30	20	26,28
5	900	25	30	20	26,33
5	1000	25	30	20	26,37
5	250	0	30	20	4,38
5	250	5	30	20	10,68
5	250	10	30	20	16,00
5	250	15	30	20	19,94
5	250	20	30	20	22,67
5	250	25	30	20	24,90
5	250	30	30	20	26,21
5	250	35	30	20	26,84
5	250	40	30	20	27,23
5	250	45	30	20	27,32
5	250	50	30	20	27,19

5	250	55	30	20	26,94
5	250	60	30	20	26,62
5	250	65	30	20	26,31
5	250	70	30	20	25,94
5	250	75	30	20	25,95
5	250	80	30	20	25,96
5	250	85	30	20	26,08
5	250	90	30	20	26,13
5	250	95	30	20	26,16
5	250	100	30	20	25,90
5	250	25	10	20	10,50
5	250	25	11	20	11,80
5	250	25	13	20	12,00
5	250	25	14	20	12,72
5	250	25	15	20	13,50
5	250	25	16	20	13,84
5	250	25	17	20	15,00
5	250	25	18	20	16,10
5	250	25	19	20	16,65
5	250	25	20	20	17,32
5	250	25	25	20	20,80
5	250	25	30	20	24,65
5	250	25	35	20	28,60
5	250	25	40	20	32,15
5	250	25	45	20	34,90
5	250	25	49	20	36,65
5	250	25	50	20	36,90
5	250	25	55	20	37,72
5	250	25	75	20	43,35
5	250	25	100	20	44,26
5	250	25	30	10	26,00
5	250	25	30	15	23,92
5	250	25	30	20	25,00
5	250	25	30	21	25,20
5	250	25	30	22	25,42
5	250	25	30	23	25,56
5	250	25	30	24	25,98
5	250	25	30	25	26,04
5	250	25	30	26	26,06
5	250	25	30	27	26,12
5	250	25	30	28	26,15
5	250	25	30	29	26,18
5	250	25	30	30	26,25
5	250	25	30	31	26,31
5	250	25	30	32	26,36
5	250	25	30	33	26,40
5	250	25	30	34	26,47
5	250	25	30	35	26,51
5	250	25	30	37	26,59
5	250	25	30	39	26,66
5	250	25	30	40	26,91

TABLE S2. Data sets extracted from the different experimental runs for the removal of TB (mg/L) by H_2O_2/periodate oxidation system under different operational conditions of initial H_2O_2 concentration (10-200 mM), initial periodate dosage (0.5-10 mM), initial solution pH (3-10.5), initial TB concentration (C_0: 5-50 mg/L) and liquid temperature (10-50 °C).

$[H_2O_2]_0$ (mM)	$[IO_4^-]_0$ (mM)	pH	Temp. (°C)	C_0 (mg/L)	TB Removal (mg/L)
10	1	5,4	25	10	5,29
20	1	5,4	25	10	5,87
30	1	5,4	25	10	6,24
35	1	5,4	25	10	6,55
50	1	5,4	25	10	7,22
55	1	5,4	25	10	6,99
60	1	5,4	25	10	6,73
70	1	5,4	25	10	6,33
75	1	5,4	25	10	6,22
80	1	5,4	25	10	5,99
85	1	5,4	25	10	5,76
95	1	5,4	25	10	5,38
100	1	5,4	25	10	5,18
110	1	5,4	25	10	4,84
115	1	5,4	25	10	4,77
125	1	5,4	25	10	4,46
130	1	5,4	25	10	4,22
135	1	5,4	25	10	4,13
140	1	5,4	25	10	3,94
150	1	5,4	25	10	3,66
155	1	5,4	25	10	3,46
160	1	5,4	25	10	3,30
170	1	5,4	25	10	2,95
175	1	5,4	25	10	2,84
180	1	5,4	25	10	2,71
190	1	5,4	25	10	2,30
195	1	5,4	25	10	2,11
200	1	5,4	25	10	2,04
50	0,5	5,4	25	10	5,5
50	0,60	5,4	25	10	6,00
50	0,70	5,4	25	10	6,33
50	0,80	5,4	25	10	6,47
50	1	5,4	25	10	6,6
50	1,30	5,4	25	10	7,02
50	1,50	5,4	25	10	7,66
50	1,70	5,4	25	10	7,90
50	1,80	5,4	25	10	8,03
50	2	5,4	25	10	8,31
50	3,00	5,4	25	10	9,13
50	3,50	5,4	25	10	9,25
50	3,70	5,4	25	10	9,31
50	4,00	5,4	25	10	9,43
50	4,50	5,4	25	10	9,48
50	5	5,4	25	10	9,51
50	5,50	5,4	25	10	9,54
50	6,00	5,4	25	10	9,39
50	7,00	5,4	25	10	9,17
50	7,50	5,4	25	10	9,09
50	8,00	5,4	25	10	8,99
50	9,00	5,4	25	10	8,78
50	10	5,4	25	10	8,66
50	1	3	25	10	8,08

50	1	3,50	25	10	7,87
50	1	3,70	25	10	7,82
50	1	4,20	25	10	7,67
50	1	4,50	25	10	7,52
50	1	4,70	25	10	7,44
50	1	5,00	25	10	7,30
50	1	5,50	25	10	6,91
50	1	5,70	25	10	6,55
50	1	6,00	25	10	5,77
50	1	6,50	25	10	5,10
50	1	7	25	10	5
50	1	7,50	25	10	3,96
50	1	7,70	25	10	3,94
50	1	8,00	25	10	3,87
50	1	8,50	25	10	3,75
50	1	8,70	25	10	3,68
50	1	9	25	10	3,7
50	1	9,50	25	10	3,43
50	1	10,00	25	10	3,13
50	1	10,20	25	10	3,03
50	1	10,70	25	10	2,78
50	1	11	25	10	3,59
50	1	5,4	10	10	7,62
50	1	5,4	12	10	7,53
50	1	5,4	13	10	7,51
50	1	5,4	16	10	7,46
50	1	5,4	17	10	7,42
50	1	5,4	19	10	7,39
50	1	5,4	20	10	7,37
50	1	5,4	22	10	7,31
50	1	5,4	24	10	7,27
50	1	5,4	26	10	7,13
50	1	5,4	28	10	6,76
50	1	5,4	29	10	6,56
50	1	5,4	30	10	6,39
50	1	5,4	32	10	6,12
50	1	5,4	33	10	6,08
50	1	5,4	35	10	5,93
50	1	5,4	36	10	5,84
50	1	5,4	37	10	5,78
50	1	5,4	40	10	5,25
50	1	5,4	42	10	4,98
50	1	5,4	44	10	4,32
50	1	5,4	45	10	3,92
50	1	5,4	46	10	3,74
50	1	5,4	48	10	3,25
50	1	5,4	50	10	2,72
50	1	5,4	25	5	4,22
50	1	5,4	25	7	5,11
50	1	5,4	25	8	5,87
50	1	5,4	25	10	7,26
50	1	5,4	25	11	7,84
50	1	5,4	25	12	8,29
50	1	5,4	25	14	9,60
50	1	5,4	25	15	9,82
50	1	5,4	25	17	11,09
50	1	5,4	25	18	11,97
50	1	5,4	25	20	13,00
50	1	5,4	25	22	14,20

50	1	5,4	25	25	15,92
50	1	5,4	25	30	18,03
50	1	5,4	25	33	18,95
50	1	5,4	25	35	19,15
50	1	5,4	25	40	20,10
50	1	5,4	25	45	20,95
50	1	5,4	25	47	21,51
50	1	5,4	25	50	22,00

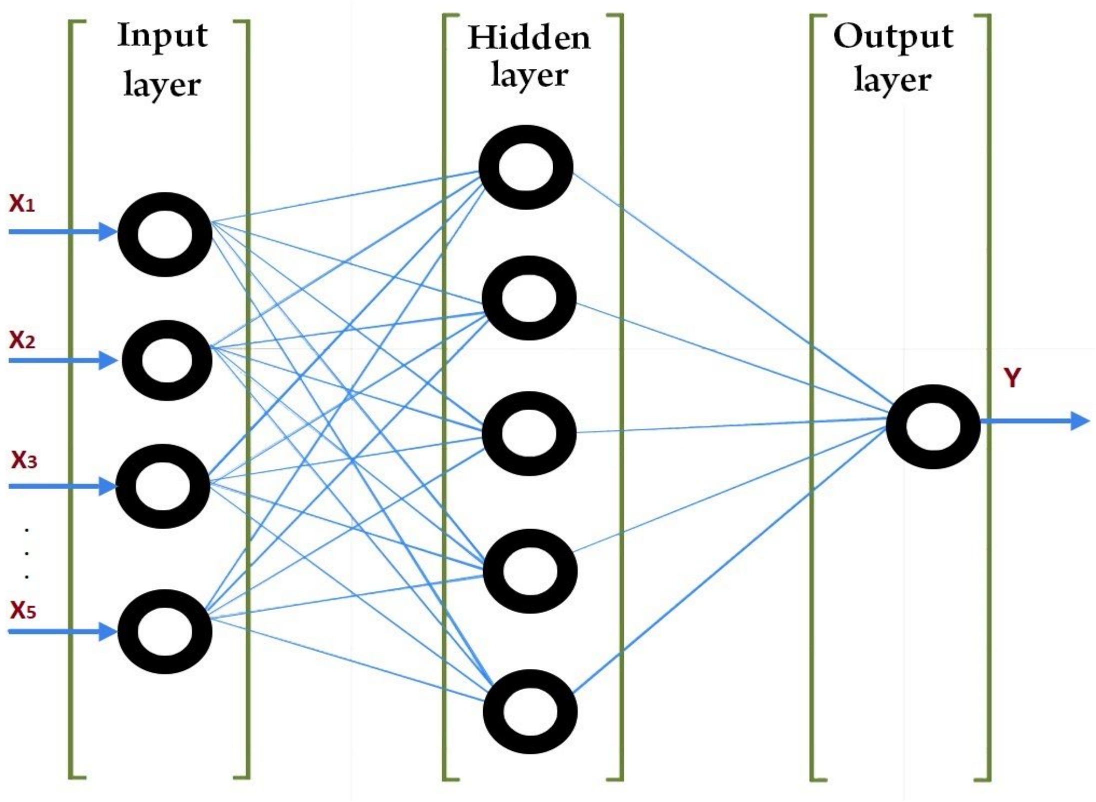

Figure. S1. Artificial neural network topology.

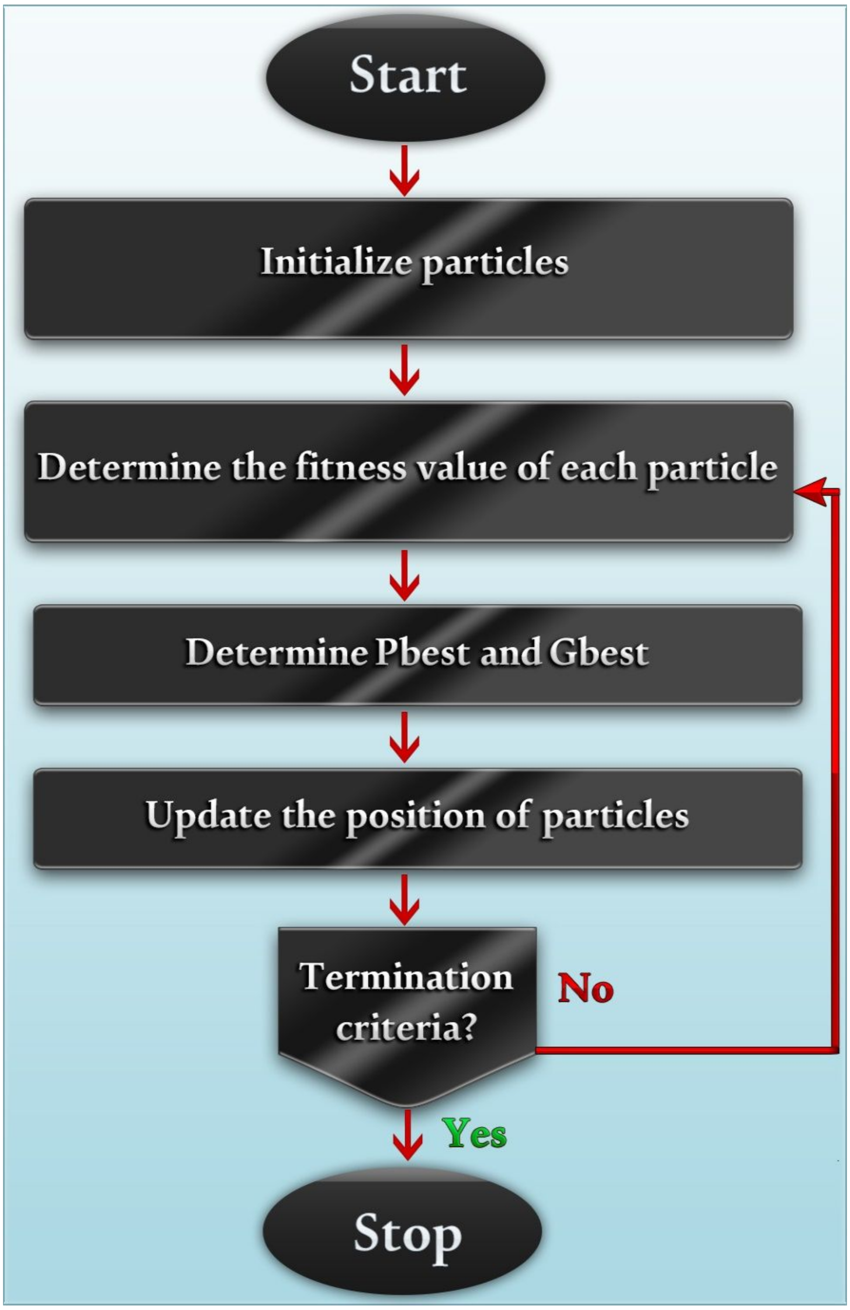

Figure. S2. Standard Flow chart of the PSO algorithm.

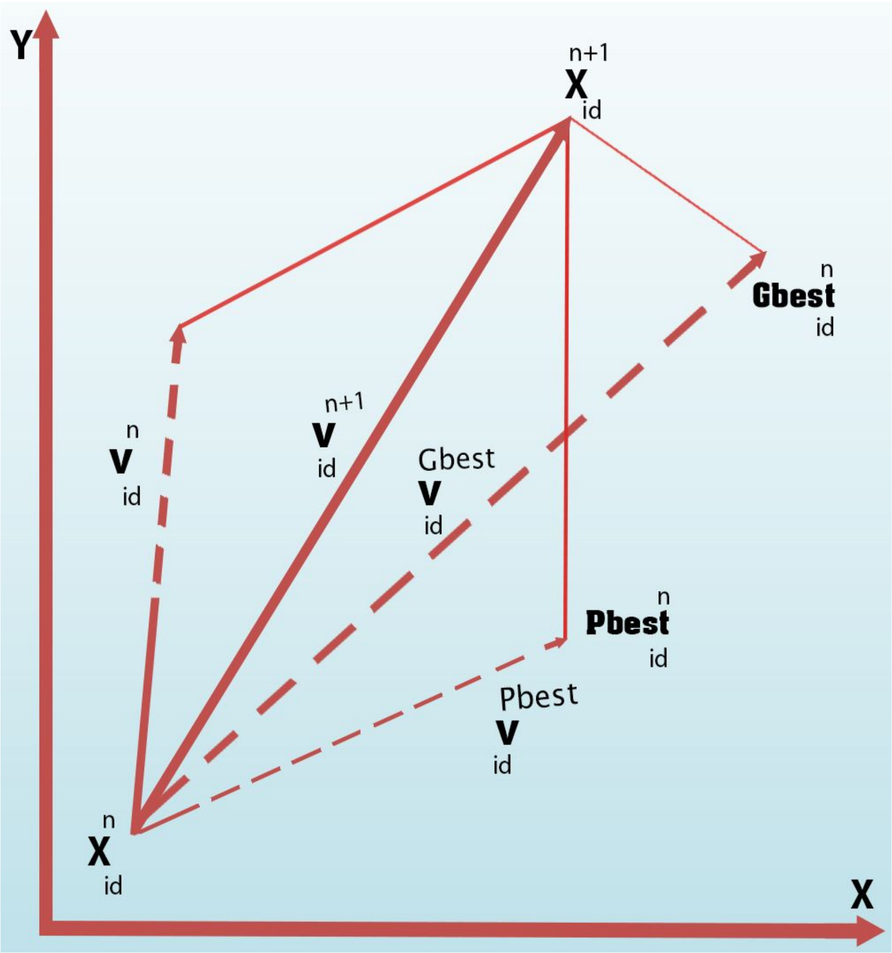

Figure. S3. Concept of changing a particle's position in PSO.

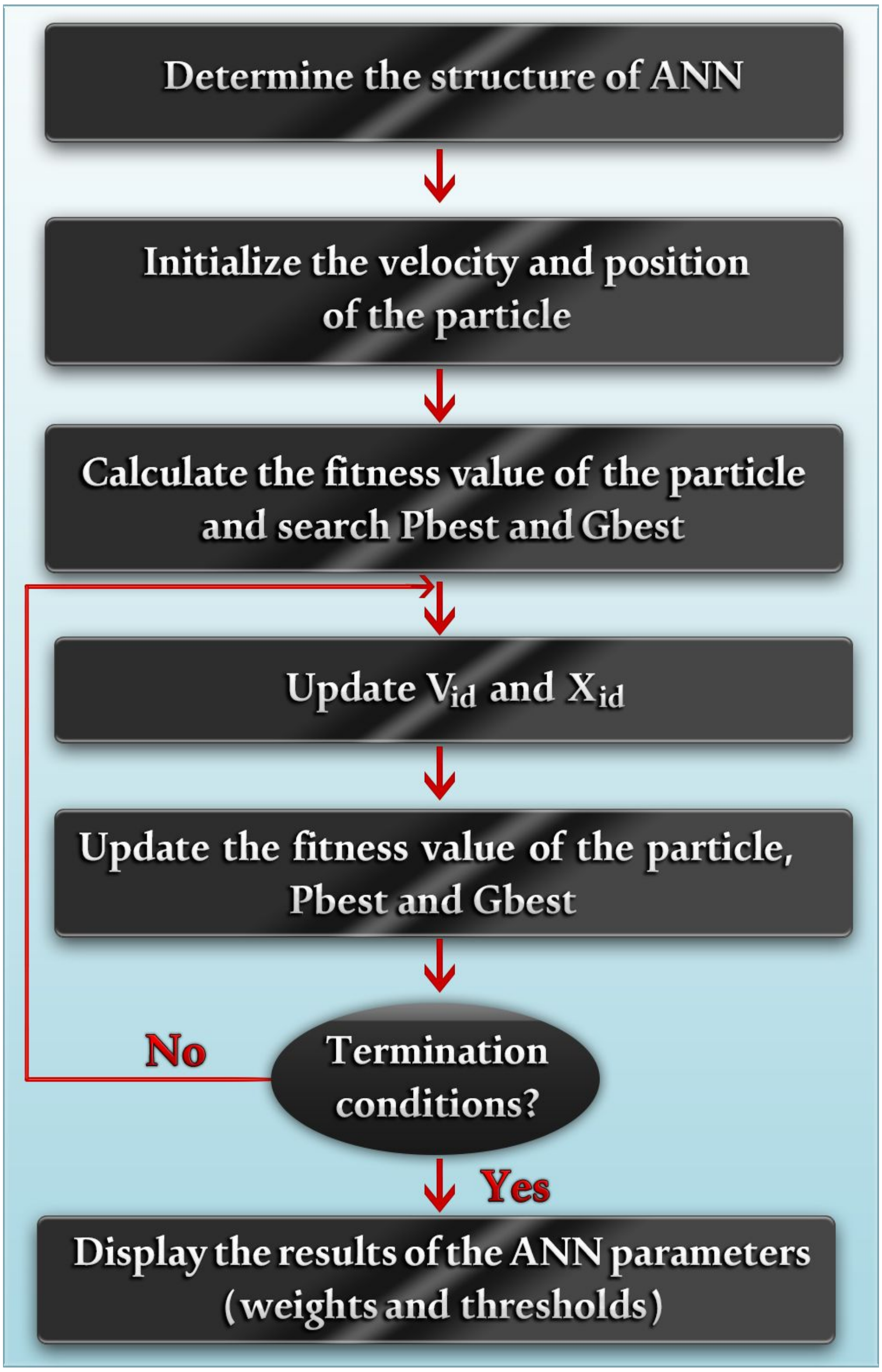

Figure. S4. Flow chart of the ANN-PSO hybrid mathematical model.
